# Commentary: Experiences and perceptions of patients, caregivers, and healthcare professionals with long-acting injectable antipsychotics for the treatment of schizophrenia: qualitative results from the multinational ADVANCE study

**DOI:** 10.3389/fpsyt.2026.1766178

**Published:** 2026-01-19

**Authors:** Yoshiyo Oguchi

**Affiliations:** Department of Neuropsychiatry, St. Marianna University School of Medicine, Kawasaki, Japan

**Keywords:** commentary, inpatient treatment, long-acting injectable antipsychotics, qualitative research, schizophrenia, shared decision-making

## 1 Introduction

The multinational ADVANCE study by Franzenburg et al. provides valuable qualitative insights into the barriers and facilitators of long-acting injectable (LAI) antipsychotic use across diverse cultural landscapes (1). By capturing the voices of patients, caregivers, and healthcare professionals (HCPs), the authors highlighted critical gaps in communication and perception. However, to fully translate these findings into clinical practice, three methodological and interpretative issues warrant careful reconsideration: the representativeness of the HCP sample, absence of triadic data, and interpretation of LAI acceptance within inpatient settings.

## 2 The risk of overgeneralization from limited samples

The study’s ambition to identify “country-level” barriers is commendable, yet the sample size requires cautious interpretation. While the study spanned eight countries, the qualitative analysis included only 17 HCPs. Broken down by nation, this resulted in only two or three clinicians representing the entire healthcare system (e.g., n=2 for Australia, Canada, Germany, Spain, and the United States; n=3 for China and Israel). Healthcare systems are profoundly heterogeneous; for instance, reimbursement policies and access to community psychiatry vary significantly even within a single country. Drawing conclusions about national trends or “global barriers” based on such a limited number of voices risks an ecological fallacy, in which the idiosyncratic views of a few clinicians are mistaken for systemic characteristics. Future research should validate these qualitative signals using larger representative cohorts to avoid stereotyping clinical practices based on small-N data.

## 3 The “missing link” in decision-making triads

A pivotal limitation acknowledged by the authors is that the patient, caregiver, and HCP cohorts were not linked and were recruited as disjointed groups. In the clinical practice of schizophrenia treatment, the decision to initiate LAI involves a dynamic interaction within this specific triad. Without linked data, it becomes difficult to accurately assess the “discordance” mentioned in the study. For example, when physicians cite “patient aversion to injections” as a primary barrier, is this an accurate reflection of their patients’ views, or is it a paternalistic assumption that preemptively shuts down the conversation? In disjointed samples, we could not verify whether a patient’s refusal correlated with their specific physician’s communication style or the caregiver’s stance. Understanding the true mechanics of shared decision making requires a study design that analyzes these three perspectives as a single interacting unit.

## 4 Reframing the inpatient setting: from barrier to opportunity

Perhaps the most critical point for clinical reconsideration is the conclusion of the study regarding care settings. The authors noted that patients treated in inpatient settings were less likely to accept LAIs that were associated with feelings of coercion, loss of control, and trauma. Although valid, this observation risks conflating the correlation with causation. It is highly probable that patients requiring hospitalization present with greater disease severity, lower insight, and higher agitation, factors that independently drive treatment resistance, regardless of the setting itself. Attributing the negative perception solely to the “inpatient environment” may obscure a vital clinical reality; the inpatient phase often represents the most secure and monitored environment for the safe transition from oral medications to LAIs. Rather than viewing hospitalization as a determinant of negative perceptions, clinicians should view it as a critical “window of opportunity.” During admission, the immediate availability of staff allows for intensive psychoeducation and the safe management of post-injection observation, potentially mitigating the very fears of “loss of control” the study identifies. If we accept the study’s implication that inpatient settings are inherently detrimental to LAI acceptance, we risk discouraging early intervention during the acute phase, particularly when LAIs could provide the greatest benefit in preventing future relapse.

## 5 Conclusion

Franzenburg et al. initiated an important dialogue on the human factors driving LAI underutilization. However, by critically examining the sample limitations, the need for triadic analysis, and the potential for reverse causality in inpatient settings, we can refine the clinical implications of their work. Moving forward, the focus should shift toward leveraging the inpatient setting as a proactive engagement point and utilizing linked data approaches to truly understand the negotiation of treatment choices.
